# Recreative Change

**Published:** 1874-09

**Authors:** 


Recreative Change.—Urged by a natural craving and a salutary custom, many medical men are now making arrangements by means of which they will be able to obtain recreation and change of thought, act and scene. Exhaustion, consequent upon continuous tension, invariably ends, sooner or later, in restlessness and irritability, and then the great vital law of change, which runs through the whole universe, asserts itself, and powerfully impels the jaded to cease from labor and shun their labor-fields. Unfortunately, during the past year we have seen in our branch of profession several melancholy examples of the fatal result of neglecting these beneficent promptings. There are some who cannot be made to heed them, and strange to say, of all offenders, those who are most successful are often the greatest. It is the old story of much would have more, and thus we find the man who has a lucrative practice and is accumulating money, the one most difficult to move. His excuses are innumerable. In vain it is pointed out to him that for his own benefit and for the sake of his family and patients, diversion is necessary. Ambition lures him on, and his brave heart battles against the warnings of failing mind and body, until at length a crisis arrives, and then that cessation from work which might have been enjoyed at a convenient season for a suitable period, is enforced, most probably at a very inconvenient time, upon a bed of pain, amidst sorrowful faces. It is sad that medical men, who ought to know better, should so often err in this way. There must be amongst them either a want of faith in the practical advantages of recreation and rest, or a wilful blindness to unmistakable indications. They would despise a farmer whose fields lack fertility because he would not grant them a fallow season, and yet they will not grant themselves that pause from labor by which alone they can gain vigor and encompass success. What appears for the time a loss, is in reality an excellent investment. The enervated man does his work ineffectively, laboriously and tardily; with restored energy he can do all with ease and enjoyment. There is, however, another great advantage in getting quite away from the scenes of our labors. It enables us to take a distant and independent view of our home position and mode of living. Amidst hills and woods and streams we can take, as it were, a bird's-eye view of our other selves, as we appear in our ordinary lives, and accurately survey our every day habits and actions. The impartial prospect thus obtained is very valuable, as it enables us to see clearly what ought to be remedied and what improved. Fresh projects flash upon the mind, and the futility of misdirected exertion becomes manifest. By rest and change alone we can recreate our effete lives and renew our lost energies. We may work in one groove until we become buried in it, our vision contracted and our intellectual life stifled. The essentiality of life is change. Immutability is death.—*The Obstetrical Journal—August.*

